# Understanding critical thinking practices in Iranian healthcare managers: Qualitative insights

**DOI:** 10.34172/hpp.025.44535

**Published:** 2025-11-04

**Authors:** Hussein Saber Hussein, Kaveh Bahmanpour, Mohammad Fathi, Adel Fatemi

**Affiliations:** ^1^Department of Health Care Management, Faculty of Medical Sciences and Technologies, Research and Sciences branch, Islamic Azad University, Tehran, Iran; ^2^Department of Nursing, Faculty of Medical Sciences, Sanandaj Branch, Islamic Azad University, Sanandaj, Iran; ^3^Department of Statistics, Faculty of Basic Sciences, Sanandaj Branch, Islamic Azad University, Sanandaj, Iran

**Keywords:** Critical thinking, Qualitative research, Health care, Health management

## Abstract

**Background:**

Critical thinking has emerged as a vital competency for effective decision-making in healthcare management, yet its conceptualization and application within culturally specific contexts remain insufficiently explored. In this qualitative study, we seek to conceptualize critical thinking within the context of Iranian healthcare management.

**Methods:**

In 2023, through conventional content analysis of semi-structured individual interviews with 17 healthcare managers from diverse roles and institutions in Sanandaj, Iran, we tried to identify key components of critical thinking within the Iranian healthcare context. The interviews lasted from 45 to 60 minutes. MAXQDA 2020 was used to manage the data.

**Results:**

From the viewpoints of our participants, the concept of critical thinking in healthcare settings means *Strategic organizational awareness*, *Adaptive leadership & staff-centered management*, *Structured decision-making*, *Operational oversight and collaboration*, and *Learning and professional development*.

**Conclusion:**

Our study provided a contextually grounded understanding of critical thinking among Iranian healthcare managers. Findings may be contributed to both theoretical and practical discourse on managerial competence in healthcare, potentially offering transferable insights for comparable global contexts. The findings can inform policy formulation, enhance professional training programs, and shape leadership strategies specific to Iran’s healthcare system.

## Introduction

 Critical thinking has emerged as a vital competency for effective decision-making in healthcare management, yet its conceptualization and application within culturally specific contexts remain insufficiently explored. While extensive research exists on critical thinking in clinical practice and Western healthcare systems,^1,2^ little attention has been paid to how healthcare managers in non-Western settings operate. Critical thinking is also an indispensable skill for healthcare leaders, enabling systematic decision-making, future forecasting, and effective organizational management to ensure safe and high-quality care.^3,4^ Additionally, recognized as a cornerstone of leadership, critical thinking provides a structured approach to evaluating employee competencies and organizational culture.^5,6^ When combined with universal skills like collaboration, problem-solving, and creativity, critical thinking enhances workforce effectiveness and fosters a productive work environment.^7,8^

 Critical thinking is often described as the application of logic, promoting clarity, open-mindedness, and intellectual rigor.^9^ Fero et al^10^ define it as reflective thinking aimed at informed decision-making, while Hussein et al^6^ expand this to include strategic planning, problem-solving, resource management, and quality oversight in healthcare settings. The evolution of critical thinking from a focus on pure logic to a teachable skill set (conceptualizing, analyzing, synthesizing, and evaluating) highlights its role in guiding actions and beliefs. ^11^ Despite its varied definitions across disciplines,^12,13^ critical thinking (CT) universally equips managers to navigate complex, dynamic challenges.^14^

 However, while the universal traits of critical thinking are well-documented, its application must adapt to specific contexts.^11^ Given the complexities of Iran’s healthcare system—including resource limitations, bureaucratic challenges, and the need for adaptive leadership—developing a contextually grounded understanding of critical thinking is crucial for enhancing managerial practices and improving organizational outcomes.

 Prior studies on critical thinking in healthcare have predominantly focused on clinical professionals, such as physicians and nurses, often neglecting the unique demands of healthcare management.^15^ Furthermore, much of the existing literature relies on quantitative assessments, which may fail to capture the nuanced, experience-based perspectives of practitioners. This study addresses these gaps by adopting a qualitative approach to explore how Iranian healthcare managers define, perceive, and apply critical thinking in their daily decision-making processes. Through in-depth interviews with key stakeholders, this research seeks to conceptualize critical thinking within the context of Iranian healthcare management, examine the role of organizational and cultural factors in shaping critical thinking practices, and identify challenges and opportunities for fostering critical thinking in Iran’s healthcare leadership.

 By illuminating these dimensions, this study advances both theoretical and practical discourse on managerial competence in healthcare. The findings could guide policy formulation, enhance professional training programs, and shape leadership strategies specific to Iran’s healthcare system, potentially offering transferable insights for comparable global contexts.

## Methods

###  Participants and Procedures

 A qualitative investigation utilizing a conventional content analysis methodology was undertaken to examine the construct of critical thinking among healthcare managers in Iran. Due to the paucity of prior research on this subject within the Iranian healthcare management context, this methodological framework facilitated the emergence of novel insights by eliciting participants’ unique viewpoints without the imposition of predetermined categories or theoretical frameworks.^16^

 The study cohort consisted of healthcare managers (n = 14), with a gender distribution of eight males and six females, ranging in age from 28 to 49 years (mean age = 37). Participants were recruited from diverse managerial roles, including hospital administration, nursing leadership, and public health management. Sampling was conducted across multiple hospitals and healthcare centers under the jurisdiction of Kurdistan University of Medical Sciences. To ensure heterogeneity, a purposive sampling strategy with maximum variation was employed, incorporating key demographic and experiential variables such as age, gender, and managerial specialization (hospital, nursing, and health management).

 Regarding academic qualifications, the sample comprised three medical specialists, three general practitioners, four individuals holding master’s degrees, and four with bachelor’s degrees. Inclusion criteria mandated a minimum of one year of managerial experience and direct engagement in supervisory roles.

###  Data Collection

 The study employed an iterative data collection and analysis process from March to July 2023 through individual in-depth interviews. These semi-structured interviews explored healthcare managers’ application of critical thinking in workplace planning, decision-making, and leadership scenarios. The first author, an experienced qualitative researcher, conducted all interviews using established protocols that began with open-ended questions (Table 1): “How would you describe critical thinking in your managerial role?” and “In what ways does critical thinking influence your decision-making processes?” This approach ensured methodological consistency while permitting natural exploration of participants’ unique perspectives.

**Table 1:** The sample questions asked during the interviews

**Main research questions**	**Probing questions**
How would you describe critical thinking in your managerial role?	Can you explain more?
In what ways does critical thinking influence your decision-making processes?	Could you elaborate on that?
What have been the most significant challenges you have faced in your role as a healthcare manager within your organization/ward/department?	Can you provide more details about those challenges?
Looking back at a particularly effective decision you made, what factors contributed to its success?	Could you elaborate on that?
What were your primary motivations and reasoning behind your chosen course of action in that situation?	Can you explain more?
What underlying assumptions or values influenced your interpretation of the situation?	Can you provide more details about those assumptions?

 Following ethical approval, participants meeting the inclusion criteria were recruited from their workplaces. Interviews occurred in private settings selected by participants, lasting 45-60 minutes to ensure comprehensive data collection while maintaining confidentiality. All audio recordings were securely stored on encrypted devices using numerical identifiers, with verbatim transcripts verified through member checking before subsequent analysis. Consistent with qualitative research standards, data collection continued until thematic saturation was achieved (after 14 interviews), with three additional confirmatory interviews conducted (total N = 17) to validate findings.

###  Data Analysis Methodology

 Interview recordings underwent verbatim transcription to generate textual datasets. The principal investigator performed iterative, in-depth readings to achieve immersion and develop a comprehensive understanding of participants’ narratives. Following initial familiarization, the textual data were systematically parsed into discrete meaning units, which were subsequently coded to identify substantive content domains. The analytical process progressed through distinct phases, including (a) primary open coding to capture fundamental elements, (b) axial categorization through comparative analysis of code similarities, and (c) thematic consolidation into higher-order conceptual frameworks. All analytical procedures were conducted using MAXQDA 2020 (VERBI GmbH) qualitative data analysis software to ensure systematic data management and enhance analytical rigor.

###  Methodological Rigor and Trustworthiness

 To ensure the credibility of the findings, this study applied Guba and Lincoln’s framework.^17^ Credibility was reinforced through: (1) purposive selection of participants with pertinent experiential knowledge and strong communicative proficiency; (2) extended engagement via iterative interviews; (3) reflexive memo documentation; (4) participant validation (member checking); and (5) peer debriefing. The research team conducted systematic verification of interview transcripts, analytical codes, thematic categories, and interpretations through an iterative review process. Discrepancies in coding or analysis were resolved via deliberative consensus. Dependability was strengthened through collaborative analysis and coding involving all research team members, ensuring integration of multiple analytical perspectives. Comprehensive documentation of contextual factors, participant demographics, and non-verbal observations further supported transferability.

 The robustness of emergent themes was validated through: (1) participant corroboration of thematic interpretations (member validation); (2) investigator triangulation via consensus-based coding validation by an expert panel; and (3) methodological triangulation integrating semi-structured interviews with non-participant observational data. This study adhered to the Consolidated Criteria for Reporting Qualitative Research (COREQ) guidelines^18^ to ensure methodological transparency and rigor across all phases of the research process. Collectively, these measures reinforced the study’s trustworthiness while maintaining systematic methodological integrity.

## Results

 Data analysis led to the identification of 197 codes, 17 subcategories, and 5 categories, illustrated in Table 2. From the viewpoints of Iranian healthcare managers, the concept of critical thinking in healthcare settings means *Strategic organizational awareness, Adaptive leadership & staff-centered management, Structured decision-making, Operational oversight and collaboration*, and *Learning and professional development*.

**Table 2:** Categories and subcategories based on the content analysis approach

**Main categories**	**Subcategories**
Strategic organizational awareness	Recognizing the characteristics, values, and missions of the organization
	Defining a clear organizational culture and specific roles aligned with the mission
	Consideration of political and governmental influences in management
	Sharing Successes
Adaptive leadership & staff-centered management	Adapting management approaches based on staff characteristics
	Understanding individual temperaments and behaviors
	Fostering positive interpersonal dynamics
	Having a multi-dimensional approach in personnel development
Structured decision-making	Evaluating problems and challenges
	Creating an efficient decision-making environment
	Developing decision frameworks
	Setting decision-making standards
Operational oversight & collaboration	Establishing supervision guidelines
	Managing information and human resources effectively
	Promoting cross-disciplinary collaboration
Learning & professional development	Designing educational strategies
	Implementing staff development programs

###  Strategic Organizational Awareness

 The healthcare managers believed that it is crucial to consider the cultural sensitivities of both staff and service consumers when making decisions. They asserted that the nature, values, and mission of each organization are unique, and healthcare managers should consider the organizational culture, and clearly define the responsibilities aligned with this culture. They also believed that without informal relationships with governmental leaders, many resources would not be allocated appropriately. As they noted, ignoring the external environmental factors, particularly political and governmental influences, may lead to significant challenges for their organizations.

 “*Efforts of the members of our parliament over the past few years have led to the installation of a PET scan in this hospital. Without his authority and special relationships with the health ministry, customs authorities, and other related organizations in Tehran, this achievement would not have been possible” *(P5).

 The participants also emphasized that decentralized decision-making, attention to the contextual motivators of staff, and adapting management styles with the traits of the staff were the most important factors contributing to their success in the workplace.

 “*In my wards, the staff have different characters and they have various cultural, financial, social, and educational backgrounds. Some are motivated by a pay increase, some are encouraged by a written letter, and others perform well without any expectations” *(P9).

###  Adaptive Leadership & Staff-Centered Management

 Analysis of meaning units derived from participants’ expressions showed that the personal and inter-personal characteristics of healthcare managers have a great influence on their performances. Risk-taking, authority, reflection, insight, knowledge of management, self-confidence, honesty, open-mindedness, openness to criticism, power for making a change, opportunism, and flexibility were the frequent personal characteristics expressed by participants.

 “*In my experience, the most effective educators have been those who provide constructive criticism. I understand that acknowledging one’s weaknesses and shortcomings can be uncomfortable; however, fostering a culture of criticism within this hospital has positively influenced my performance. I consistently consider feedback from my staff, including those who may criticize me unjustly, and I reflect upon their perspectives, which occasionally leads to personal and professional growth. I experienced this over and over again” *(P4).

 They also represented that effective interpersonal communication, collaboration, teamwork, maintaining dynamism in groups, empathy, explanatory power, and the ability to manipulate minds and thoughts as important interpersonal features of healthcare managers.

 “*Sometimes when one of my staff is absent unexpectedly and I have to find another person, I know. This makes me proud because they often say: “we are coming only for your request”*. *In this situation, I know I have been able to make a good relationship” *(P7).

###  Structured Decision-Making 

 The expressions of participants showed that challenges are vital for the survival and development of an organization. They believed that a comprehensive understanding and scientific analysis of organizational problems, the ability to prioritize challenges, and being aware of lead-lag relationships are crucial tasks for healthcare managers.

 “*As a manager, I usually encounter hundreds of problems in early mornings. Some of these issues are similar to those I faced yesterday, while others are completely new. My staff expects me to solve these problems magically on my own, but in reality, that’s impossible. Thus, I must prioritize these problems effectively” *(P11).

 They also stated that there is more than one solution to each problem. As participants reported, intermediate managers, staff, and even our customers can participate in the problem-solving process by proposing different solutions. Each group has a unique perspective on organizational challenges, which results in the generation of multiple solutions. These alternatives should be organized, and ultimately, the best intervention should be implemented. However, a few top healthcare managers believed that in certain situations, the manager should make the decision independently.

 “*Sometimes the proposed solutions are hugely exceeded. I always take the time to consider all the alternatives. While this can be a time-consuming and sometimes frustrating process, I start by grouping the options based on their similarities and differences. This process continues through a selection stage, deciding which alternatives to keep and which to discard” *(P2).

###  Operational Oversight & Collaboration

 A majority of participants reported that without considering a series of clear criteria, decision-making will be useless. The most frequently mentioned criteria were “reliance on accurate knowledge and information”, “an explainable cost-benefit ratio”, “acceptability by both staff and patients”, “practical feasibility in the field”, “having a focus on goals”, “minimal conflicts of interest”, “alignment with external environmental conditions (political, economic, and cultural factors), and “hesitancy in decision-making”.

 “*Decisions are made to achieve positive outcomes. As a manager, I often assess the potential positive and negative outcomes before making decisions, as well as the human and financial resources that will need to be allocated. Today, it is easier to evaluate the risks associated with decisions. Some decisions are not worthwhile because they require excessive resources” *(P13).

 The participants acknowledged the crucial role of establishing supervision guidelines in the workplace. They noted the existence of numerous valid tools that contain precise items and clear criteria that can be used as benchmarks for this purpose. Participants specifically referred to criterion-referenced tools recommended for performance appraisal, including the goals and objectives outlined in the organization’s strategic planning, accreditation standards, quality assurance components, and upstream documents provided by the health ministry.

 “*In my opinion, the provided checklists, especially those derived from the context of Iranian hospitals, such as accreditation forms, are very comprehensive. However, there is an interesting paradox: in a majority of completed evaluations, the results are not aligned with the perceptions of the recipients of the services. Therefore, I believe there is a serious need for developing sensitive and specialized tools that utilize real criteria” *(P7).

 Our participants also discussed how the emergence of infectious diseases, particularly the COVID-19 pandemic, served as a pivotal moment for them. They emphasized the importance of multidisciplinary cooperation, effective information management in a virtual environment, human resource management, making critical decisions, and sharing achievements as additional responsibilities for healthcare managers during this crisis.

 “*During the COVID-19 crisis, we experienced significant staff attrition. Some staff members passed away, while many of our best nurses and healthcare professionals left their positions for various reasons. This shortage was particularly pronounced in treatment departments. To address this problem, we decided to recruit students. However, their lack of experience created challenges for us” *(P10).

 “*An experienced manager understands that relying on routine approaches during a crisis is ineffective and that alternative methods must be employed to manage unusual circumstances. When we requested government officials to implement a complete shutdown, they were very upset. They were concerned about potential public protests, as the existing sanctions had severely affected people’s livelihoods. Eventually, they consented to our request” *(P4).

###  Learning & Professional Development

 Participants emphasized the crucial role of skilled and dedicated staff in organizational success. They noted that a primary responsibility of healthcare managers is to develop manpower effectively. To achieve this goal, they emphasized both short- and long-term empowerment plans, which may include assessing the educational needs of personnel, delegating responsibilities, implementing retraining programs, identifying talents within the organization, and hiring staff based on their expertise.

 “*Unfortunately, one of the most significant challenges in hospital management is the improper delegation of duties. I believe that everyone is suited for specific tasks, and it is the manager’s responsibility to identify those tasks. In hospitals, we often see service personnel without formal educational training who have a strong desire to transition into nursing assistant roles. Sometimes, I face considerable pressure to accommodate this” *(P5).

 They also emphasized that multi-dimensional development—encompassing ethical, professional, and social aspects—should be prioritized and that managers should create opportunities for step-by-step progression for their staff.

 “*In some health organizations, staff members grow like mushrooms. Political events have resulted in the appointment of one hundred managers overnight!. The lack of knowledge and experience, combined with arbitrary practices based on trial and error among new managers, leads to the decline of the organization” *(P3).

## Discussion

 This study explored critical thinking practices among Iranian healthcare managers, revealing a multi-faceted understanding of this competency that extends beyond mere problem-solving. Our findings indicate that critical thinking in this context encompasses strategic organizational awareness, adaptive leadership, structured decision-making, operational oversight, and a commitment to learning and professional development. These dimensions align with the broader conceptualization of critical thinking as a vital competency for effective decision-making in healthcare management, enabling systematic decision-making, future forecasting, and effective organizational management to ensure safe and high-quality care.^19^ Critical thinking in healthcare management is primarily regarded as a skill focused on problem-solving. It offers professional healthcare leaders the ability to effectively address patients’ issues. ^19^ In a previous study, Geng^20^ studied 64 descriptions of critical thinking and concluded that scholars use judgment, argumentation, questioning, information processing, problem-solving, metacognition, skills, and dispositions as core components of critical thinking. He also asserted that the disciplinary context of scholars directly influences their perspectives on this concept. Similar to our findings, Haase^21^ demonstrated that many indicators and features of critical thinking align with various stages of the decision-making and problem-solving processes in management.

 The emphasis on strategic organizational awareness highlights the importance of contextual understanding in applying critical thinking. Managers in our study recognized the need to consider cultural sensitivities, organizational values, and even political influences in their decision-making. This resonates with the idea that while universal traits of critical thinking exist, its application must adapt to specific contexts.^22^ As Gyenes^11^ argues, critical thinking is not a one-size-fits-all approach but requires adaptation to the specific environment. In another study, Atkinson^23^ also noted critical thinking as a social and cultural practice. Although there are widely accepted definitions of critical thinking, educators and practitioners often feel the need to create a personal or working definition that suits their specific circumstances.^11^ This is particularly relevant in Iran’s healthcare system, which faces unique challenges such as resource limitations and bureaucratic complexities.

 Adaptive leadership and staff-centered management emerged as a key component of critical thinking in our study. The participants emphasized the importance of personal and interpersonal characteristics, such as risk-taking, open-mindedness, empathy, and effective communication. Hester^24^ also highlighted a mindset that includes adopting different perspectives, seeking potential, managing ambiguity, focusing on growth, and being goal-oriented as essential traits that leaders should develop. The research conducted by Khajevand et al^5^ demonstrated that communication, information sharing, education, learning, media literacy, risk-taking, and tolerance for ambiguity positively influence the development and enhancement of critical thinking in managers.These findings support the notion that critical thinking is closely linked to leadership effectiveness,^26^ providing a structured approach to evaluating employee competencies and organizational culture. Furthermore, the emphasis on collaboration, teamwork, and maintaining dynamism in groups aligns with the World Economic Forum’s (2018) assertion that critical thinking, when combined with skills like collaboration and problem-solving, enhances workforce effectiveness.^7^

 Our study also revealed that Iranian healthcare managers utilize structured decision-making processes, emphasizing the importance of understanding organizational problems, prioritizing challenges, and considering multiple solutions. This aligns with the definition of critical thinking as applied logic, promoting clarity, open-mindedness, and intellectual rigor. The participants’ focus on criterion-based decision-making, including reliance on accurate knowledge, cost-benefit analysis, and acceptability by stakeholders, underscores the importance of a systematic and reflective approach to decision-making, as highlighted by Fero et al.^10^

 The findings related to operational oversight and collaboration emphasize the practical application of critical thinking in ensuring quality and efficiency. The participants acknowledged the importance of establishing supervision guidelines, managing information effectively, and promoting cross-disciplinary collaboration. This reflects the role of critical thinking in strategic planning, problem-solving, resource management, and quality oversight in healthcare settings.^27,28^

 Finally, the emphasis on learning and professional development underscores the dynamic nature of critical thinking. The participants recognized the importance of developing staff competencies through empowerment plans, retraining programs, and talent identification. This aligns with the concept of critical thinking as a teachable skill set that can be developed through conceptualizing, analyzing, synthesizing, and evaluating information.^29,30^ Tripathy^31^ noted that critical thinking is crucial for personal development and workplace management, as it shapes the actions, decisions, and choices we make.Bittner and Gravlin^32^ also highlighted that critical thinking enhances delegation, interpersonal communication, and teamwork among healthcare managers. Zori and Morrison^8^ demonstrated that improving critical thinking skills and dispositions among nurse managers can foster positive work environments for staff registered nurses. When registered nurses work in an environment perceived as positive, they are better positioned to deliver high-quality and safe patient care. All these findings along with those we identified also reflect the understanding that in today’s rapidly changing healthcare landscape, continuous learning and adaptation are essential for effective management.

## Conclusion

 In conclusion, our study provides a contextually grounded understanding of critical thinking among Iranian healthcare managers. By highlighting the importance of strategic organizational awareness, adaptive leadership, structured decision-making, operational oversight, and learning and professional development, this research contributes to both theoretical and practical discourse on managerial competence in healthcare, potentially offering transferable insights for comparable global contexts. The findings can inform policy formulation, enhance professional training programs, and shape leadership strategies specific to Iran’s healthcare system.

## Competing Interests

 The authors declare no conflict of interest.

## Data Availability Statement

 Data are available upon reasonable request. The data that support the findings of this study are available from [Vice Chancellor for Research], but restrictions apply to the availability of these data, which were used under license for the current study, and so are not publicly available. Data are however, available from the authors upon reasonable request and with permission of [Vice Chancellor for Research].

## Ethical Approval

 This study received ethical approval from the Institutional Review Board of Islamic Azad University, Sanandaj Branch (Ethics Code: IR.IAU.SDJ.REC.1402.030; Approval Date: 01/07/2023). Prior to participation, all subjects were fully informed about the study objectives and methodology, after which written informed consent was obtained. Participants explicitly authorized audio recording of interviews through separate consent procedures. To maintain participant confidentiality, all audio files were de-identified by assigning unique numerical codes and removing personally identifiable information. Access to raw data was strictly limited to authorized research personnel throughout the study duration.
